# Reliability and Validity of the Urdu Version of Psychosomatic Symptoms Scale in Pakistani Patients

**DOI:** 10.3389/fpsyg.2022.861859

**Published:** 2022-04-11

**Authors:** S. Mudasser Shah, Muhammad Jahangir, Wei Xu, Yonggui Yuan

**Affiliations:** ^1^Department of Psychosomatics and Psychiatry, Zhongda Hospital, School of Medicine, Southeast University, Nanjing, China; ^2^Department of Psychiatry and Mental Health, Second Xiangya Hospital, Central South University, Nanjing, China

**Keywords:** psychosomatic symptoms, Urdu, gender difference, PSSS scale, SCL scale, somatization

## Abstract

The current study was aimed to assess the reliability and validity of the Urdu version of the Psychosomatic Symptoms Scale (PSSS) in Pakistani patients. The PSSS is time-saving and easy to administer. The field experts drafted the translated version of PSSS. The Urdu version of PSSS, Patient Health Questionnaire- 9 (PHQ-9), and Symptom Checklist-90 (SCL-90) Urdu version were used for assessment. The translation procedure was comprised of three steps, namely forward translation, back translation, and expert panel discussion. A sample of 982 (men = 50.5% and women = 49.5%) was collected with a convenient sample technique from a general hospital and private clinic. The Cronbach’s alpha for PSSS was 0.974. The confirmatory factor analysis (CFA) revealed that all the items factor loading of PSSS were more than 0.35, the root mean square error of approximation (RMSEA) was = 0.062, the standardized root mean residual (SRMR) was = 0.043, and the comparative fit index (CFI) was = 0.97 with 90% CI. The results also showed that women (*M* = 72.08, *SD* = 6.79) are more likely to have psychosomatic symptoms than men (*M* = 51.21, *SD* = 13.36) on *P* < 0.001. The PSSS Urdu version is proven to be a useful and reliable instrument for screening, monitoring, and assessing Pakistani patients’ psychosomatics symptoms.

## Introduction

In 1818, a German psychiatrist named Heinroth, coined the word “Psychosomatic,” and Felix Deutsch then later coined the word with the extension “Psychosomatic medicine” in 1922. The word “Psychosomatic” is derived from Greek words “Psyche” and “soma,” which mean mind and body, respectively. The term psyche originally meant “soul or mind,” but it has subsequently been referred to as behavior. In contrast, soma denotes the organism’s body Psychosomatic refers to a wide range of concepts, from illnesses to biopsychosocial research and consultation-liaison work (Chandrashekar and Math, 2006; Shamim, 2012; Dhimole et al., 2016). According to the biopsychosocial model (Engel, 1977), it refers to the connection of psychological and biological factors that are associated with the concept of health and disease. This paradigm entails the idea that physical and psychological conditions are not separated in psychosomatic assessments. Moreover, individuals with psychosomatic diseases are focused on their somatic symptoms generally ignoring their psychological difficulties (Engel, 1977; Bauer et al., 2011; Bolton and Gillett, 2019). Clinicians only rely on the easily administered tool Psychosomatic Symptoms Scale (PSSS) to assess and diagnose the psychosomatic symptoms which was developed by Li et al. (2020). PSSS measures both the aspects of psychological and somatic problems, which provided adequate support to psychologists, clinicians, and psychiatrists in making precise diagnoses and characterizing other features affecting treatment. Moreover, the scale can convey a baseline for finding progress in illness during follow-up over a period in answer to a particular intervention (Shafique et al., 2017; Li et al., 2020).

In Pakistan, the national language is Urdu, and a massive fraction of the population cannot read, write, or speak English. There are few culturally adopted scales in the Urdu language used to diagnose and assess psychosomatics. In clinical settings and for research purposes, Symptoms Check List (SCL-90) is used widely, which is a multidisciplinary and lengthy scale with 90 items that is rather time-consuming. A short, reliable, and easily administered tool is required to assess the patients (Gaynes et al., 2008). Other than the SCL-90, the PSSS is very easy to administer in general hospitals and clinical setups. The PSSS is a shorter and valid scale with 26 items, which is time-saving for both the patients and doctors. However, the PSSS is only available in English and Chinese language. Therefore, the urge was felt to translate and adapt the PSSS in the Urdu language. The Urdu version of this scale will be filled by patients themselves without assistance for translation and will be handy for both clinicians and researchers. Moreover, the Urdu version of this scale will aid in avoiding misdiagnosis or inappropriate diagnosis. Therefore, the main objective of the current study was to provide a valid and reliable Urdu version of the PSSS scale to doctors, psychologists, and researchers.

## Materials and Methods

Informed consent was received from patients before filling out the questionnaires. The present study was approved by the ethical committee of the hospital (2021ZDSYLL347-P01). In phase 1, the PSSS scale was translated into the Urdu language, and in phase II, the psychometric properties of the PSSS scale were calculated. The confirmatory factor analysis (CFA) was used to assess the factorial structure of two dimensions of PSSS. The correlation was inspected among the PSSS, SCL-90, and Patient Health Questionnaire- 9 (PHQ-9).

### Measures

#### The Psychosomatic Symptoms Scale

The PSSS was used to measure the symptoms of distress that occurred in the last 7 days. There scale has 26 total items, with each item rated and valued on a 4-point scale from not at all rated (0) to all the day rated (Shamim, 2012); the scale usually takes 15 min to complete. It was used to measure two main dimensions of symptoms, namely psychological and somatic complaints. The psychological symptoms were evaluated by 5, 10, 11, 12, 17, 21, and 25, while somatic symptoms were estimated by 1, 2, 3, 4, 6, 7, 8, 9, 13, 14, 15, 16, 18, 19, 20, 22, 23, 24, and 26 items of the scale (Li et al., 2020).

#### The Symptom Checklist-90

The Symptom Checklist-90 (SCL-90) has a 5-point Likert scale, with nine subscales of phobic anxiety, somatization, depression, obsessive-compulsive, paranoid ideation, interpersonal sensitivity, hostility, psychoticism, and anxiety. The reliability of SCL-90 is.98 (Shafique et al., 2017). This scale is used in many languages (Imperatori et al., 2020; Bianciardi et al., 2021). We used the subscale somatization Urdu version. The Urdu version of SCL-90 comprised of 90 items and used to determine criterion validity.

#### The Patient Health Questionnaire-9

The Urdu version of Patient Health Questionnaire-9 (PHQ-9) was used in the present study as a standard measure. PHQ-9 has 9 items, and each item measures depression and starts from 0 to 3. The reliability of PHQ-9 is 0.91 (Ahmad et al., 2018).

### Phase I: Translation of Psychosomatic Symptoms Scale Into the Urdu Language

The translation of PSSS was carried out in four main steps. The first step involved the translation of PSSS from the English language to the target language “Urdu,” with the help of four experts in the relevant fields (one psychiatry professor, one psychology professor, one Urdu literature Ph.D. scholar, and one English literature scholar). All the experts were requested to translate the PSSS questionnaire word by word without changing the meaning.

The second step involved determining the discrepancies, and assessment of the items was conducted under the supervision of the four subject experts (clinical psychologist, a professor, an associate professor, an assistant professor, and one senior researcher); each item was assessed thoroughly, with choosing the grammar and proper words near to the original one in Urdu.

The third step involved the Brislin method for back-translation. For that purpose, four experts (two English language professors, one psychology professor, and one Urdu professor) were requested to accurately translate the PSSS Urdu version into the English language. The scale was applied on 30 sample sizes with a mean age of 24.5 years (*SD* = 5.2). The results proved no ambiguity in the questionnaire and were ready for further validation (Tanku, 2013).

### Phase II: To Determine the Psychometric Properties of Psychosomatic Symptoms Scale

To quantify the psychometric properties of PSSS, the Cronbach’s alphas were calculated. CFA was used to accomplish the construct validity of PSSS in AMOS 23 (Arbuckle, 2014).

### Participants

Participants were selected for research from the psychiatric clinic (Dr. A. Sadiq Psychiatric clinic, Peshawar, Khyber Pakhtunkhwa) and general hospital (Hayatabad Medical Complex, Peshawar Khyber Pakhtunkhwa) before COVID-19. The participants were asked to fill out the questionnaires manually. The inclusion criteria were the following: inpatients and outpatients from the general hospital and clinic, with age ranging from 20 to 65 years old.

### Statistical Analysis

The data were analyzed by using IBM SPSS version 22 and AMOS version 23 (IBM Corp, 2013; Arbuckle, 2014). The Cronbach’s alpha of PSSS was primarily measured. Spearman’s correlation test was applied to find the association of PSSS with PHQ-9 and SCL-90. *P*-value (*P* < 0.05) was considered significant for all the statistical analyses. Data from the sample of 982 patients were used to verify the item characteristics, factor and structure validities, and internal consistency of PSSS. Mean and *SD*, as well as corrected item-total correlation (CITCs), were measured for each item in the scale. CITCs values greater than 0.3 are considered good (Cristobal et al., 2007). Item number 20 of the PSSS measures different symptoms depending on genders, such as premature male ejaculation and female dysmenorrhea or irregular menstruation. Spearman’s correlation was used for the age factor. Construct validity was computed by using Spearman’s correlation between PSSS, SCL-90, and PHQ-9. CFA was used to find the factorial validity. The model, estimation of weighted least squares mean and variance (WLSMV), was used. The root mean square error of approximation (RMSEA) and comparative fit index (CFI) with 90% CI. For testing the model fit, the standardized root mean residual (SRMR) was used. The model fit indicated TLI > 0.95, CFI > 0.95, SRMR < 0.08, and RMSEA < 0.06.

## Results

The data were taken from 982 diagnosed patients with psychiatric disorders from different departments, as shown in Table 1. The questionnaires were filled in, and the basic demographic characteristics were obtained for the current study, including sex, education, age, employment status, financial status, marital status, and department.

**TABLE 1:** Demographics of participants.

	Variable	Frequency	Percentage
Sex	Male	496	50.5
	Female	486	49.5
Age	20–29	482	49.1
	30–39	431	43.8
	40–49	62	6.40
	50–59	7	0.7
Education	High school	579	58.9
	Undergraduate education	189	19.2
	Graduate education	214	21.9
Marital status	Married	762	22.2
	Unmarried	220	77.8
Job	Employed	554	56.4
	Unemployed	423	43.1
	Retired	5	0.5
Financial Status	Low	376	38.3
	Medium	412	41.9
	High	194	19.8
Department	Psychiatry	168	17.1
	Oncology	152	15.4
	Endocrinology	143	14.6
	Gynecology	140	14.2
	Cardiology	85	8.7
	Urology	80	8.2
	Orthopedic	58	6.0
	Hematology	53	5.4
	Dermatology	38	3.8
	General Surgery	35	3.6
	Maxillofacial	30	3.0

*PSSS, Psychosomatics Symptoms Scale, Low = less 4,000–much 20,000, Middle = 50,000–100,000, High = 100,000 and above (Status, 2022).*

### Reliability and Characteristics of All Items of Psychosomatic Symptoms Scale

Reliability coefficients and descriptive statistics (*N* = *982*) were computed for PSSS. The results were tabulated and given in Table 2.

**TABLE 2:** Psychometrics properties of major study variables.

					Range			
Variables	N	M	SD	*Alpha*	Max	Min	Skew	Kurt
PSSS	982	61.54	14.89	0.974	26	78	−0.684	−0.676

*PSSS, Psychosomatic Symptoms Scale; Skew, skewness; Kurt, kurtosis.*

Table 2 shows the psychometric properties of the scale PSSS. The Cronbach’s α value for PSSS is 0.974, indicating the scale’s high reliability. The maximum range is 26, while the minimum range is 78. The mean for total participants (*N* = 982) is 6.54, and the *SD* is 14.89. The value of skewness = −0.684 and Kurtosis = −0.676 are negative. As far as skewness and kurtosis are concerned, the PSSS is in the range of ± 1. The scale as a whole has negative values. The negative values show that data has the highest values, while the positive shows that data has the lowest. According to Kim (2013), if values of skewness are less than 2 and the value of kurtosis are less than 7 for any sample (*N* > 300), it can be considered as the normal distribution (Kim, 2013) (shown in Table 2).

### Internal Consistency of Psychosomatic Symptoms Scale Urdu Version

Table 3 shows all the items of PSSS, their mean, standard deviation, and corrected item- Total Correlations CITCs. Range of response options covered by the responses to each item. The mean CITCs of items ranged from 0.437 to 0.897. In the given table, most of the items have high Cronbach’s alpha, this high Cronbach’s alpha shows good and strong reliability.

**TABLE 3:** Internal consistency of Psychosomatic Symptoms Scale (PSSS) Urdu version.

S/No	Items	Mean (SD)	Corrected item- total correlations	Cronbach’s Alpha if item deleted
1	Dizziness	2.41 (0.708)	0.734	0.973
2	Eye discomfort	2.29 (0.672)	0.778	0.973
3	Burning or tight sensation	2.44 (0.688)	0.723	0.972
4	Limb trembling or numbness	2.24 (7.05)	0.759	0.973
5	Depressed mood	2.24 (0.839)	0.644	0.974
6	Cardiovascular symptoms	2.39 (0.698)	0.850	0.973
7	Pulmonary symptoms	2.45 (0.684)	0.831	0.974
8	Discomfort of throat	2.41 (0.698)	0.808	0.973
9	Tinnitus	1.37 (0.739)	0.830	0.973
10	No interest	2.35 (0.766)	0.640	0.972
11	Anger or irritation	2.35 (0.762)	0.662	0.974
12	Nervousness or panic	2.33 (0.770)	0.668	0.974
13	Dryness of mouth	2.39 (0.741)	0.846	0.973
14	Sour regurgitation	2.36 (0.750)	0.860	0.972
15	Nausea or vomiting	2.40 (0.725)	0.837	0.974
16	Abdominal symptoms	1.43 (0.678)	0.847	0.972
17	Avoidance of anxiety	2.32 (0.787)	0.674	0.974
18	Urine abnormality	2.37 (0.832)	0.859	0.972
19	Perineal discomfort	2.38 (0.953)	0.891	0.973
20	Sexual (male) or menstrual (female) dysfunction	2.38 (0.753)	0.897	0.972
21	Suicidal thoughts	2.27 (0.756)	0.437	0.971
22	Feeling heat or cold	2.39 (0.773)	0.852	0.972
23	Pain	2.33 (0.695)	0.700	0.974
24	Tiredness	2.30 (0.753)	0.769	0.974
25	Repeated thoughts or actions	2.33 (0.834)	0.672	0.972
26	Sleep difficulties	2.36 (0.723)	0.762	0.973

*PSSS, Psychosomatic Symptoms Scale.*

### Mean Differences Across Gender

Mean differences along gender were computed by independent *t*-test. The results were generated was then tabulated and displayed in Table 4.

**TABLE 4:** Mean differences across gender on PSSS.

Measures	Male (*n* = 496)		Female (*n* = 486)		*t*(498)	*P*	95% CI	
				
	*M*	*SD*	*M*	*SD*			LL	UL
PSSS	51.21	13.36	72.08	6.79	−30.75	0.001	−22.20	−19.54

*PSSS, Psychosomatic Symptoms Scale. P < 0.001.*

Table 4 revealed significant differences of psychosomatic symptoms in men and women. The scores of women were higher on PSSS (*M* = 72.08, *SD* = 6.79) compared to men (*M* = 51.21, *SD* = 13.36) on *p* < 0.001.

### Validity of Psychosomatic Symptoms Scale

The sum score of PSSS was significantly correlated with that of PHQ-9 (*r* = 0.589, *P* < 0.001) and SCL-90 subscale somatization (*r* = 0.635, *P* = 0.001), showing the good construct validity. Some items had moderate to high correlation of SCL-90.

### Factor Validity of the Psychosomatic Symptoms Scale

**FIGURE 1:**
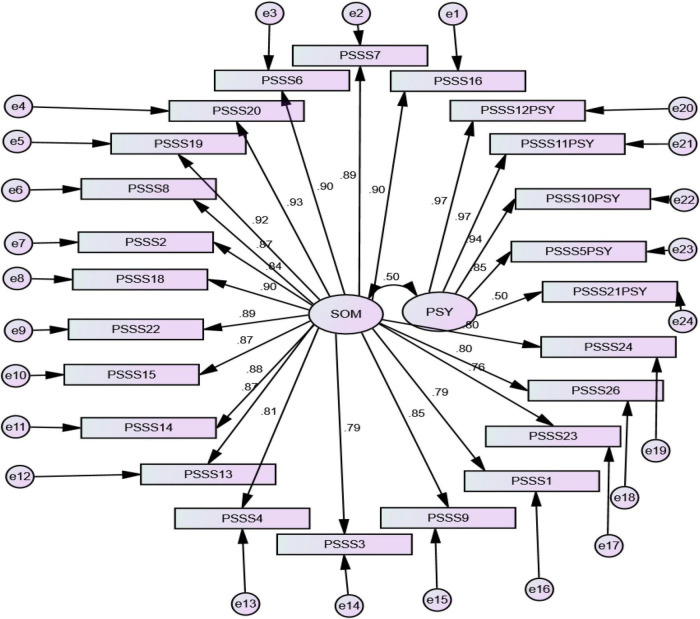
The confirmatory factor analysis of two subscales of Psychosomatic Symptoms Scale (PSSS). Som, somatic complaint; Psy, psychological complaints.

Confirmatory factor analysis explained the underlying dimensions and the relationship between the observed and original factors. Exploratory factor analysis (EFA) was also used to find the validity of the scale. The Kaiser-Meyer-Okin (KMO) test of sphericity by component analysis was 0.98 and the Bartlett’s spherical test result was 325 (*P* < 0.000). These values showed that EFA could be accepted. CFA is a statistical technique used to verify the factor structure of a set of observed variables. In initial conditions, the standardized regression weights of all the observed factors that were high than 0.35 would be acceptable (Field, 2013). The model fit was good with RMSEA = 0.062, SRMR = 0.043 which is excellent, and the CFI = 0.97 which is also an excellent value. In the current study, all factors had higher CFA than standard value 0.35 except item no. 17 and 25. It proved that the factor models of the two subscales P and S. The factor loadings looked at the trail of observed pointers with covariance. All the values were greater than 0.35 as shown in Figure 1.

## Discussion

The current study was aimed to validate and translate the PSSS into the Urdu language. PSSS is the first scale for the assessment of patients measuring both psychological symptoms and physical complaints and their association because physical symptoms frequently occurred with psychological distress (Li et al., 2020).

Pakistan has more than 200 million human population, and the mental health is poorest but there are less than 500 psychiatrists serving this population. This low number of mental health professionals creates a big treatment gap, which is why more than 90% of the common mental disorders remain untreated (Bashir, 2018; Sikander, 2020). The needs for the care of individuals suffering from mental illnesses have been documented in this region for many centuries. Indigenous healthcare systems, such as Unani and Ayurveda treatment models, had a considerable influence on healthcare over the decades. The religious and cultural ideas about indigenous and traditional healthcare were always visible in local systems. They significantly impacted choices, awareness, and perception for several health-related treatments (World Health Organization [WHO], 2009).

Public health sectors are the key spots for mental health problems, but recently, private sectors have contributed to mental health treatment (Ali and Gul, 2018). Gender-based analysis for PSSS revealed that women score high on the P and S subscale of PSSS, wherein the score represents that women show severe symptoms compared to patients who are men. Previous studies reported that women rated higher for somatic symptoms than men (Bardel et al., 2019), supporting our results. However, in Pakistan, the literacy rate among women is relatively lower than men, which possibly be the cause of women scoring high on PSSS. Most of the patients are unaware of mental health, wherein they go to various clinics and hospitals for a checkup which diagnose them with various diseases. Due to a large number of patients, the psychiatrists cannot provide the deserving duration of time to diagnose or educate the patients about their illness. The patient’s attendants were unaware of the illness and frequently checked their patient with various medical professionals. Misdiagnosis of the patient, which leads to the wrong prescription of medicine, may progressively exacerbate the disease. Other reasons are additional responsibilities of women, such as caring for children and their housekeeping duties (Kishi et al., 2002; Scott et al., 2006; Bashir, 2018; Sikander, 2020). Also, the studies suggest a negative impact of conflict between the physical and mental wellbeing of women (Bourbonnais et al., 1999; Artazcoz et al., 2004). In this study, men have fewer psychosomatic complaints than women. Previous research reported that women have higher psychosomatic symptoms compared to men (Östberg et al., 2006; Brooks et al., 2021), which supports our results for translated versions of PSSS.

Therefore, the PSSS Urdu version is a valid and reliable tool for psychosomatics, and it could be used in Urdu-speaking communities for convenient and precise diagnosis.

## Conclusion

In conclusion, our study demonstrated that the PSSS Urdu version is a useful and reliable instrument for screening, monitoring, and assessing Pakistani patients’ psychosomatics symptoms. The lower educational level of women compared to men, the reliance on spiritual healers, and the lack of health literacy might contribute to the gender difference in the prevalence of symptoms.

### Significance of the Study

The current study will contribute to the convenient diagnosis of psychosomatic disorder. It is also easy to administer in general hospitals in Pakistan. This scale is short and time-saving and will aid further research.

### Limitation and Suggestions

The PSSS scale was applied only to the patients of province KP-Pakistan. To generalize its findings, it is mandatory to use this scale to various populations of Pakistan. Second, PHQ-9 was used instead of PHQ-12 and PHQ-15 because only PHQ-9 was available in the national language (Urdu language). Applying new and updated scale of PHQ-9 is therefore suggested.

## Data Availability Statement

The datasets presented in this study can be found in online repositories. The names of the repository/repositories and accession number(s) can be found in the article/Supplementary Material.

## Ethics Statement

The studies involving human participants were reviewed and approved by the Ethical Committee of Zhongda Hospital, Southeast University, Nanjing, Jiangsu, China (2021ZDSYLL347-P01). The patients/participants provided their written informed consent to participate in this study.

## Author Contributions

SS and YY: conceptualization and methodology and data analysis. SS: writing original draft preparation. YY, WX, and MJ: review. All authors contributed to the article and approved the submitted version.

## Conflict of Interest

The authors declare that the research was conducted in the absence of any commercial or financial relationships that could be construed as a potential conflict of interest.

## Publisher’s Note

All claims expressed in this article are solely those of the authors and do not necessarily represent those of their affiliated organizations, or those of the publisher, the editors and the reviewers. Any product that may be evaluated in this article, or claim that may be made by its manufacturer, is not guaranteed or endorsed by the publisher.
