# Supplementary material for: Reliability and Validity of the Urdu Version of Psychosomatic Symptoms Scale in Pakistani Patients
Source: Front Psychol. 2022 Apr 11;13:861859. doi: 10.3389/fpsyg.2022.861859 (PMC9037750; doi:10.3389/fpsyg.2022.861859)
Supplement: Supplementary file 1 [file Table_1.DOCX]

**PSYCHOSOMATIC SYMPTOM SCALE (PSSS)**

**نام_____________ عمر _________________ تعلیم ______________**

**صنف _____________ ماہانہ آمدن ______________**

**شادی شدہ/غیرشادی شدہ ___________________** پیشہ ____________________

مندرجہ ذیل فہرست ان مسائل اور شکایات کےبارے میں ہے جو لوگوں کو اکثر پیش آتے ہیں۔ہر ایک کو غور سے پڑھیں۔ ایسا کرنے کے بعد کسی ایک کیفیت کو منتخب کریں جیسے مثال کے طور پر کسی پریشانی یا مسئلے نے آپکو پچھلے ہفتے حتیٰ کے آج بھی الجھائے رکھا ہے۔ دائیں جانب دیئے گئے مسلےپر ہندسے میں دائر ہ لگائیں اور کسی بھی جز کو خالی نہ چھوڑیں۔

| "0"پر دائرہ لگائیے، اگر آپ کا جواب ہے**،"بالکل نہیں**" |
| --- |
| "1" پر دائرہ لگائیے، اگر آپ کا جواب ہے " بہت کم " |
| "2" پر دائرہ لگائیے، اگر آپ کا جواب ہے" آدھے دن سے زیادہ " |
| "3"پر دائرہ لگائیے، اگر آپ کا جواب ہے" سارا دن یا زیادہ " |
|  |

| سارا دن یا زیادہ | آدھے دن سے زیادہ | بہت کم | بلکل نہیں | *پچہلے چار ھفتوں میں سے آپ کتنے دفعہ نیچے دی گیں علامات میں سے گزرے ہیں*  *() کا نشان لگائیں* | نمبر شمار |
| --- | --- | --- | --- | --- | --- |
| 3 | 2 | 1 | 0 | چکر آنا یا سر کا بھاری محسوس ہونا | 1 |
| 3 | 2 | 1 | 0 | آنکھیں خشک یا تکلیف یا دھندلاپن | 2 |
| 3 | 2 | 1 | 0 | جسم میں جلن یا تنگی کا احساس ہونا | 3 |
| 3 | 2 | 1 | 0 | ہاتھ پاؤں کا ہلنا یا سن ہونا | 4 |
| 3 | 2 | 1 | 0 | افسردہ یا نا امید ہونا | 5 |
| 3 | 2 | 1 | 0 | سینے میں تکلیف یا دھڑکن کاریز ہونا | 6 |
| 3 | 2 | 1 | 0 | سینے کی جکڑنا، خرخراہٹ یا سانس میں تکلیف | 7 |
| 3 | 2 | 1 | 0 | گلے میں تکلیف محسوس کرنا | 8 |
| 3 | 2 | 1 | 0 | کانوں یا دماغ میں بھنبھناھٹ | 9 |
| 3 | 2 | 1 | 0 | خوشی کے کاموں میں دلچسپی نہ لینا یا معنی خیز نہ لگنا | 10 |
| 3 | 2 | 1 | 0 | معمول سے زیادہ غصہ یا چڑچڑا لگنا | 11 |
| 3 | 2 | 1 | 0 | گھبراہٹ، پریشانی یا خوفزدہ ہونا | 12 |
| 3 | 2 | 1 | 0 | منہ خشک ہونا یا زبان کا بھاری پن | 13 |
| 3 | 2 | 1 | 0 | ڈکار، کھٹی کھجلی یا معدے میں جلن | 14 |
| 3 | 2 | 1 | 0 | متلی یا قے ہونا | 15 |
| 3 | 2 | 1 | 0 | پیٹ میں گڑگڑاھٹ، درد، قبض یا اسہال | 16 |
| 3 | 2 | 1 | 0 | ایسے صورتحال سے کنارہ کشی کرنا جو خفہ یا گھبراھٹ پیدا کرے | 17 |
| 3 | 2 | 1 | 0 | پشاب میں تکلیف یا بار بار آنا | 18 |
| 3 | 2 | 1 | 0 | عجان میں تکلیف | 19 |
| 3 | 2 | 1 | 0 | قبل از وقت انزال یا جران (مردوں)  غیرمعمولی حیض (عوتوں) | 20 |
| 3 | 2 | 1 | 0 | اپنے آپ کو تکلیف دینے کے خیالات آنا | 21 |
| 3 | 2 | 1 | 0 | بخار یا سردی لگنا | 22 |
| 3 | 2 | 1 | 0 | کسی خاص جگہ میں درد یا سارے جسم میں | 23 |
| 3 | 2 | 1 | 0 | سستی کا تھکاوٹ کا احساس | 24 |
| 3 | 2 | 1 | 0 | بار بار ایک کام کرنا یا خیالات آنا | 25 |
| 3 | 2 | 1 | 0 | سونے میں دشواری، آسانی سے بیدار ہونا یا نیند نہ آنا | 26 |
| Total Score: | | | | |  |

The author got the permission for usage and translation of PSSS from Dr. Yonggui Yuan Contacat Dr. Yonggui Yuan(yygylh2000@sina.com) Or S. Mudasser Shah for using Urdu version of PSSS
